# Unusual presentation of ‘numb chin syndrome’ as the manifestation of metastatic adenocarcinoma of the lung^☆^

**DOI:** 10.1016/j.ijscr.2013.08.024

**Published:** 2013-09-25

**Authors:** Faizan Zaheer, Khurrum Hussain, Jeethendra Rao

**Affiliations:** a University of Manchester, Room 1.03, Coupland 3 Building, Coupland Street, Manchester M13 5PL, United Kingdom; b Heaton Mersey Dental Practice, Heaton Mersey, Stockport SK4 3BY, United Kingdom; c Manchester Royal Infirmary, Oxford Road, M13 9WL, United Kingdom

**Keywords:** Numb chin syndrome, Mental neuropathy, Brain metastasis

## Abstract

**INTRODUCTION:**

Numb chin syndrome (NCS) is the presence of hypoaesthesia or paraethesia of the lip and chin over the distribution of the mental nerve. It is often caused by the presence of a metastatic tumour in the mandible or the base of skull and represents advanced malignancy.

**PRESENTATION OF CASE:**

This paper presents an unusual case of NCS associated with metastatic adenocarcinoma of the lung, for which no obvious lesion was found in the mandible or base of the skull.

**DISCUSSION:**

NCS can oftentimes present itself in the absence of mandibular or base of skull metastatic lesions.

**CONCLUSION:**

NCS can be a sign of underlying advanced metastatic malignancy and therefore cannot be ignored and must be investigated fully.

## 1 Introduction

“Numb chin syndrome” (NCS), sometimes also referred to as “mental neuropathy”, is a sensory neuropathy characterised by hypoesthesia, paraesthesia or less commonly pain over the chin in the region supplied by the mental nerve and its branches.^1,2^ It was first described by Charles Bell in his monograph of 1830 “the nervous system of the human body”, when a lady with breast cancer presented with insensibility of one side of the lower lip. Further investigation showed the presence of a hard glandular mass on the jaw which was most likely pressing against the inferior alveolar nerve and causing the numbness.^3^ Subsequently in 1963 Calvery et al. coined the phrase “numb chin syndrome”.^4^ This can be a very ominous sign as it may indicate tumour metastasis to the mandible and can often be the first manifestation of systemic malignancy.

## 2 Presentation of case

A 62-year-old lady was referred by her General Physician to the maxillofacial department regarding numbness of the lower left lip and chin present for the past 3 months. She also complained of increased sensation on the left half of her tongue and an alteration in taste to the left anterior two-thirds of the tongue along the distribution of the left lingual nerve.

Her co-morbidities included spinal stenosis and neuralgia paraesthetica (left lateral cutaneous nerve in thigh) that was being managed by analgesics and gabapentin. She quit smoking 30 years ago. She smoked a total of less than 5 pack years of smoking.

On examination there were no signs of local dental pathology. An orthopantomogram X-ray showed no significant findings and due to ongoing concern an urgent MR scan of the head and neck area was requested.

Nine days after this presentation the patient attended the Emergency Department and was admitted complaining of a sudden onset of left lateral pleuritic chest pain associated with shortness of breath. A working diagnosis of pulmonary embolism was made while awaiting the results of a CT Pulmonary Angiogram. This revealed a mass in the apical segment of the left lower lobe with a pleural effusion, most likely to be malignant, and multiple lesions in the liver.

**Fig. 1:**
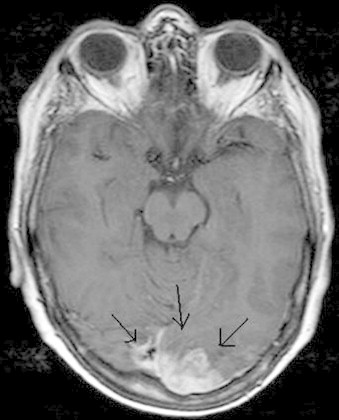
A 3 cm wide left occipital metastatic mass with oedema of the white matter.

**Fig. 2:**
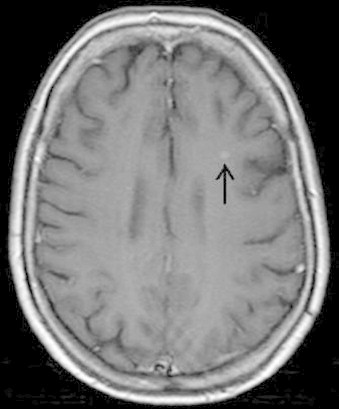
Small metastatic lesion in the left frontal lobe.

Pleural aspirate cytology confirmed the presence of cells consistent with an adenocarcinoma. The results of the MR scan of the head were now available showing a 3 cm wide left occipital mass with oedema of the white matter and another small lesion in the left frontal lobe (see Figs. 1 and 2). This was thought most likely to be a metastasis from her lung carcinoma. Her lung tumour was staged as a T4 N1 M1b, (stage IV) with probable brain metastasis. Subsequent bone scans also confirmed bony metastatic disease in both her legs.

Her symptoms of numb lip and chin were managed with gabapentin which was already prescribed and she was referred for further management to a chest physician and clinical oncologist. Palliative radio- and chemotherapy was offered, however, the patient refused treatment. There was a general deterioration of the patient's condition and the patient died 4 and a half months after her initial diagnosis of numb chin syndrome.

## 3 Background

In the literature the reported aetiology of NCS is widely ranging. Local pathological processes such as trauma, infections, tumours and cysts are amongst common causes.^5^ Iatrogenic factors such as nerve damage following ID blocks, third molar extractions and root canal therapy can often also present as numbness of the chin and lip.^6–8^

Immune mediated systemic conditions such as temporal arteritis, vasculitis, multiple sclerosis Sjogren's syndrome have all been linked with NCS.^5,9–11^ Reports of NCS in sickle cell crisis have also been published with some cases presenting with bilateral numbness.^12–14^ Other causes include connective tissue disease, systemic infections, toxic exposures, nerve root entry zone plaques, vertebro-basilar insufficiency and brain stem infarcts.^15^

Malignancy is one of the most prevalent causes of NCS^2^ and can represent widespread metastatic disease often being its first presenting sign.^1,2,11,15,16^ In more than 30% of cases it can precede relapse or recurrence.

Pathogenic mechanisms behind NCS are thought to be either infiltration or compression of the inferior alveolar nerve sheath by tumour tissue. This can take place either at the level of the mandible or more proximally along the mandibular nerve near the base of the skull.^1,2,15^

A study of 42 patients in 1992 showed that the most common malignant cause of NCS was breast cancer or lymphoproliferative neoplasms. They comprised 78% of malignancy associated NCS cases with 50% of NCS cases as a result of mandibular metastasis, 22% as a result of leptomeningeal seeding and 14% as a result of base-of-skull lesions.^17^

Prognosis of patients with NCS as a result of malignancy is very poor with survival being measured in months.^1,2^ Median survival following diagnosis can be as little as 5 months if NCS is caused by bone metastasis and 12 months if associated with leptomeningeal seeding.^17^ Treatment of NCS is provided by treating the underlying cause and management of symptoms. In the case of malignancy, management is usually palliative due to very poor prognosis.^15^

NCS is purely a sensory neuropathy usually presenting as unilateral numbness or paraesthesia over the distribution of the mental nerve.^2^ Pain and swelling may occur if there is a superimposed infection or advanced local destruction by malignancy. Generalised symptoms of malignancy such as weight loss, fever, localised pain and night sweats may also be present.^2,16^ The mean time of onset of NCS in cancer patients is 4 years after their primary cancer diagnosis.^17^

NCS cannot be taken lightly. It is important to recognise the potential for sinister underlying pathology associated with unilateral chin or lip numbness.^2,15^ Current expert opinion is that NCS of unknown cause is to be treated as suspected malignancy until proven otherwise.^18^

Panoramic radiography of the jaw can provide valuable information. Any metastatic or local tumours can often be seen as well as any other causative local dental pathology. Further detailed views can be obtained through CT imaging. Imaging of the brain and skull base can also be very useful as it may reveal proximal lesions affecting the mandibular division of the trigeminal nerve. Some authors suggest the use of MRI if other forms of imaging are unrevealing.^2^

## 4 Discussion

In the case presented imaging did not show any evidence of mandibular metastasis or involvement of the base of skull. It was unlikely that compression by the occipital lobe metastasis was the cause of NCS, because one would expect this to cause other cranial nerve deficits.^19^ Therefore it is possible that there may be microscopic seeding of malignant cells within the nerve not visible on imaging. Since symptoms of neuropathy of the lingual nerve were also seen, it is likely that infiltration of the proximal aspect of the mandibular division of the trigeminal nerve had occurred close to the skull base, at a level before the posterior trunk divides into the lingual and inferior alveolar nerves.

There have been several other case reports of NCS where there has been no evidence of a metastatic lesion in the mandible or base of skull on imaging.^18,20^ It is important to remember that lack of radiographic changes cannot exclude the possibility of small metastatic lesions in the jaw.^21^ Friedrich reported a case of tumour infiltration of the inferior alveolar nerve inside the mandibular canal that was found on surgical exploration after a negative CT scan. Laurencet et al. presented a case of small cell carcinoma of the lung with NCS and no signs of an infiltrative lesion in the brain or mandible. One month later a metastatic tumour appeared on the mandible. This supports the theory that microscopic infiltration of the nerve can elicit symptoms of NCS before the tumour becomes clinically detectable.^19^

An important question is why are metastatic tumours selective for the mandible and mandibular nerve? Bodner et al. suggest that there may be a selective aspect in the site of seeding of metastatic tumours which may be responsible for the mandibular predilection in metastasis. Other authors have suggested that due to the presence of rich red bone marrow posterior mandible is rendered more susceptible to tumour embolisation.^22^ All of these explanations are hypothetical and poorly understood.

## 5 Conclusion

NCS can be a sign of underlying advanced metastatic malignancy and therefore cannot be ignored. It must be investigated fully with the help of plain film and high resolution cross sectional imaging as appropriate for each case. It is possible that metastatic tumours infiltrating the inferior alveolar nerve may not be evident on imaging and can pose added difficulty in diagnosis.

## Conflict of interest

None declared.

## Funding

None.

## Ethical approval

No ethical approval was required for this paper and I can confirm that I have consent from next of kin (son) as the patient has passed away.

## Author contributions

Faizan Zaheer (lead author): played a role in patient management and the lead author of writing up the case report.

Khurrum Hussain: aided in writing up the case report and proof reading.

Jeethendra Rao: clinical lead in patient management and aided in writing up the case report and proof reading.
